# Diagnosis of Acute Pancreatitis Secondary to Hypertriglyceridemia in a Pediatric Patient in the Emergency Department

**DOI:** 10.7759/cureus.33403

**Published:** 2023-01-05

**Authors:** Kyle R Tomasini, Dakota K Tomasini, Brit Long

**Affiliations:** 1 Emergency Medicine, Brooke Army Medical Center, Fort Sam Houston, USA; 2 Pediatrics, Brooke Army Medical Center, Fort Sam Houston, USA

**Keywords:** lipoprotein lipase deficiency, abdominal pain, pediatric pancreatitis, emergency department, hypertriglyceridemia induced pancreatitis

## Abstract

The incidence of acute pancreatitis in pediatric patients has increased in the last decade. We present the case of an eight-year-old boy who presented to the emergency department from another healthcare facility for the evaluation of appendicitis and was found to have acute pancreatitis secondary to hypertriglyceridemia. Clinical suspicion of pancreatitis should remain high in the pediatric patient population with nausea, emesis, and abdominal pain considering that pancreatitis’ often atypical and non-specific presentation may lead to delayed diagnosis.

## Introduction

The incidence of pancreatitis in the pediatric patient population has become increasingly common in recent decades, reaching the level of adults [1]. Despite an incidence rate of approximately 13.2 per 100,000 [1], few published case reports have been presented on the early identification of pancreatitis in the emergency department (ED) and the differences in the presentation of the disease in children versus adults. While pancreatitis in adults is typically secondary to gallstones and alcohol use [2], the etiology among pediatric patients is more extensive, including drugs, infection, trauma, genetic disorders, anatomic abnormalities, and autoimmune disorders [1]. The clinical presentation in pediatric patients is often non-specific with symptoms such as emesis, irritability, and non-specific abdominal pain. Although heightened clinical suspicion and subsequent increase in lipase testing have played a significant role in appropriate diagnosis [1], a gap still remains in promptly identifying a pediatric patient with acute pancreatitis in the ED setting given the variability of underlying etiologies, often ambiguous presentation, and the variable volume of pediatric patients at different healthcare facilities.

## Case presentation

A previously healthy eight-year-old male patient presented to the ED via ambulance with four days of generalized abdominal pain. The patient’s mother reported associated decreased oral intake and several episodes of non-bloody, non-bilious emesis. The patient was checked at an outpatient urgent care facility earlier in the day and diagnosed with constipation with subsequent successful bowel movements after the administration of oral magnesium citrate. However, his abdominal pain persisted and migrated focally to his right lower quadrant, which prompted a return to care at another treatment facility prior to transfer to the ED for appendicitis rule-out. He received 300cc lactated ringers, ondansetron, morphine, and acetaminophen prior to arrival at the ED. During the physical examination, the patient complained of right lower quadrant pain with tenderness at McBurney’s point, rebound tenderness with palpation of the left lower quadrant, and pain upon jumping on his right leg; all of these findings were consistent with the diagnosis of appendicitis. There was no pain in the mid-epigastric region. Laboratory assessment was significant for hyponatremia (124mEq/L), leukocytosis (13.8mg/dL), elevated lipase (282U/L), and c-reactive protein (16.73mg/L).

**Figure 1:**
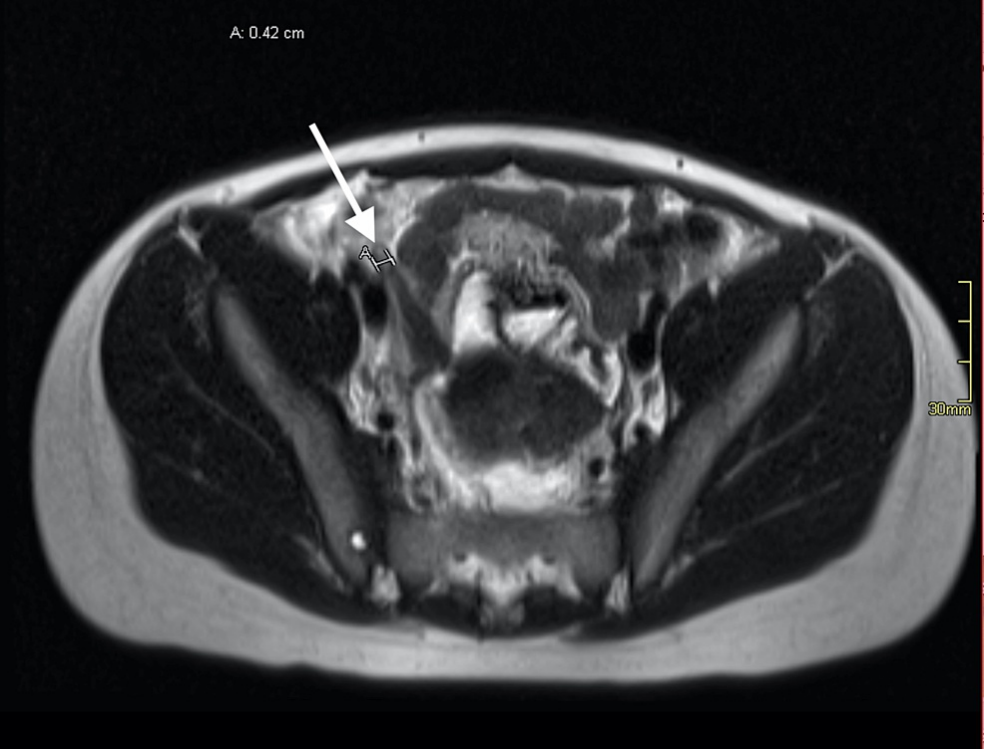
MRI of the abdomen revealing free fluid throughout the peritoneum, retroperitoneum, and extraperitoneal pelvis.

**Figure 2:**
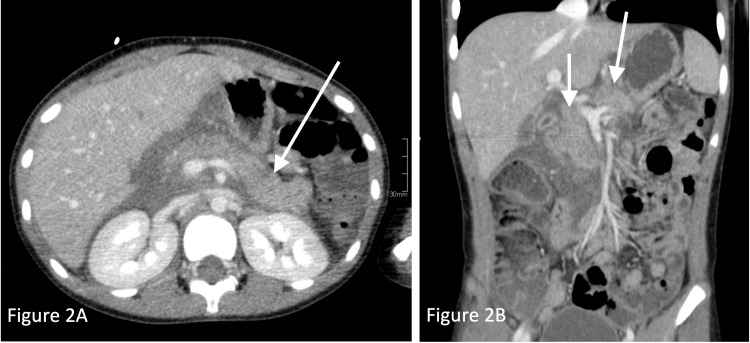
CT abdomen and pelvis with IV contrast revealing pancreatic enlargement, peripancreatic fluid, and intraperitoneal and retroperitoneal fluid, raising concerns for acute pancreatitis.

The appendix could not be visualized on limited right lower quadrant ultrasound. Following the hospital’s protocol, a fast acquisition magnetic resonance imaging (MRI) was subsequently performed; the imaging revealed a normal-appearing appendix but indicated concern for an extensive inflammatory process with free fluid throughout the peritoneum, retroperitoneum, and extraperitoneal pelvis (Figure 1). Given that the origin of this extensive inflammatory process could not be identified on fast acquisition MRI, a computer tomography (CT) of the abdomen and pelvis with intravenous contrast was recommended, and the findings were consistent with acute pancreatitis. Specifically, the CT showed pancreatic enlargement, peripancreatic fluid, and intraperitoneal and retroperitoneal fluid (Figure 2A and Figure 2B).

The patient was admitted to the pediatric ward for the management of acute pancreatitis and euvolemic hyponatremia in the setting of gastrointestinal losses. His treatment consisted of supportive measures through IV fluids with slow correction of his hyponatremia and pain control with Tylenol. He was noted to have hypertriglyceridemia at 622mg/dL on admission and was subsequently treated as an outpatient for presumed lipoprotein lipase (LPL) deficiency. He did not require insulin at any point in his treatment course, and his triglycerides slowly normalized without a pharmacotherapy intervention. He had a normal MRCP as an outpatient with pending genetic testing at the time of this write-up.

## Discussion

We present a case of acute pancreatitis in a pediatric patient; on initial presentation, it was believed that the patient was experiencing constipation, which was followed by a diagnosis of appendicitis. While previous reports have presented various etiologies for appendicitis, few have highlighted the unique challenges of initial diagnosis in an ED setting and the differences in the disease’s presentation in the pediatric population versus the adult population [3,4].

The diagnosis of pancreatitis requires the presence of at least two of the following symptoms: mid-epigastric abdominal pain, serum amylase or lipase values greater than three times the upper limit of normal, and radiologic evidence of findings consistent with pancreatitis [5,6]. In this case, two of these three criteria were met, with the lipase being more than three times the upper limit (282U/L)-a normal lipase level for an eight-year-old child is 4.0-39.0U/L [6]-and CT findings being consistent with acute pancreatitis. Further, elevated lipase is not exclusive to the diagnosis of acute pancreatitis and can be seen in cholecystitis, intestinal obstruction, peptic ulceration, diabetic ketoacidosis, celiac disease, human immunodeficiency virus, and renal insufficiency [1].

Abdominal pain is a common presenting symptom of acute pancreatitis in pediatric patients, with 62-89% of these patients experiencing localized epigastric pain and 12-20% experiencing diffuse abdominal pain [1] In this case, there was no epigastric tenderness with palpation on physical examination, and the physical examination of the patient indicated a concern for appendicitis.

The first-line choice of imaging modality for the diagnosis of pancreatitis is an abdominal ultrasound to visualize the pancreas. Although not typically needed, if these findings are equivocal, CT with IV contrast must be considered [1,6]. When there is clinical suspicion of appendicitis, a limited right lower quadrant ultrasound may be performed for certain patient subgroups, including pediatric patients. In this presentation, a complete ultrasound of the abdomen may have revealed concerns for pancreatitis, which were later revealed with CT. The ultrasound of the abdomen would have limited the child’s exposure to radiation during CT imaging, in addition to the long-term complications associated with radiation. Additionally, ultrasound serves as a low-cost and time-sensitive alternative to MRI.

The etiology of acute pancreatitis in children is more extensive than that of adults, with hypertriglyceridemia reported in one to four percent of the cases [7]. Typically, triglyceride levels greater than 1,000 mg/dl are found to result in acute pancreatitis, which is most commonly the result of lipoprotein lipase (LPL) deficiency in this population [8]. LPL is responsible for the hydrolysis of triglycerides from chylomicrons and very low-density lipoprotein. Failure of this cascade results in the accumulation of free fatty acids and chylomicrons, increasing blood viscosity, which, in turn, induces inflammation of the pancreatic tissue [9]. In this case, our patient only had hypertriglyceridemia at 622 mg/dL, although this is still the likely cause of his acute pancreatitis since he was found to have LPL deficiency. The clinical severity and rate of complications in acute pancreatitis due to hypertriglyceridemia are often higher as compared to any other cause, making prompt diagnosis essential in preventing recurrence [8].

Lastly, although the differential diagnosis for abdominal pain in a child is extensive, anchoring on the well-known and widely used criteria for the diagnosis of pancreatitis may run the risk of missed diagnosis of pancreatitis in pediatric patients given the disease’s often non-specific or atypical presentation, as was demonstrated in this case. Thus, acute pancreatitis should be retained on the differential diagnosis for children presenting with nausea, emesis, and abdominal pain. Our patient was checked at three different healthcare facilities before the diagnosis of pancreatitis was made, highlighting the importance of providing strict return precautions in an ED setting when discharge is deemed appropriate.

## Conclusions

The incidence of acute pancreatitis in pediatric patients has increased in recent decades. The diagnosis of acute pancreatitis in a pediatric patient in an ED setting can be challenging given the disease’s vast variability of etiologies and often ambiguous presentation, which varies from that of adults. In this case, an eight-year-old male patient presented to the ED of a healthcare facility from another for appendicitis rule-out; he was found to have acute pancreatitis secondary to hypertriglyceridemia. The clinical severity and rate of complications in acute pancreatitis secondary to hypertriglyceridemia are often higher in comparison to other etiologies, making prompt diagnosis essential in preventing recurrence.
